# Ecological interventions to prevent and manage zoonotic pathogen spillover

**DOI:** 10.1098/rstb.2018.0342

**Published:** 2019-08-12

**Authors:** Susanne H. Sokolow, Nicole Nova, Kim M. Pepin, Alison J. Peel, Juliet R. C. Pulliam, Kezia Manlove, Paul C. Cross, Daniel J. Becker, Raina K. Plowright, Hamish McCallum, Giulio A. De Leo

**Affiliations:** 1 Hopkins Marine Station, Stanford University, Pacific Grove, CA 93950, USA; 2 Woods Institute for the Environment, Stanford University, Stanford, CA 94305, USA; 3 Department of Biology, Stanford University, Stanford, CA 94305, USA; 4 Marine Science Institute, University of California, Santa Barbara, CA 93106, USA; 5 National Wildlife Research Center, USDA-APHIS, Fort Collins, CO 80521, USA; 6 Environmental Futures Research Institute, Griffith University, Nathan, Queensland 4111, Australia; 7 South African DST-NRF Centre of Excellence in Epidemiological Modelling and Analysis (SACEMA), Stellenbosch University, Stellenbosch 7600, South Africa; 8 Department of Wildland Resources and Ecology Center, Utah State University, Logan, UT 84321, USA; 9 US Geological Survey, Northern Rocky Mountain Science Center, Bozeman, MT 59715, USA; 10 Department of Microbiology and Immunology, Montana State University, Bozeman, MT 59717, USA; 11 Department of Biology, Indiana University, Bloomington, IN 47403, USA

**Keywords:** ecological interventions, cross-species transmission, management, spillover, zoonotic diseases

## Abstract

Spillover of a pathogen from a wildlife reservoir into a human or livestock host requires the pathogen to overcome a hierarchical series of barriers. Interventions aimed at one or more of these barriers may be able to prevent the occurrence of spillover. Here, we demonstrate how interventions that target the ecological context in which spillover occurs (i.e. ecological interventions) can complement conventional approaches like vaccination, treatment, disinfection and chemical control. Accelerating spillover owing to environmental change requires effective, affordable, durable and scalable solutions that fully harness the complex processes involved in cross-species pathogen spillover.

This article is part of the theme issue ‘Dynamic and integrative approaches to understanding pathogen spillover’.

## 1. Introduction

Pathogen spillover, or the transmission of infections among species, can occur from animals to humans (zoonoses), from humans to animals (reverse zoonoses), or even from abiotic environmental reservoirs into vertebrates (sapronoses). Environmental change—including deforestation, habitat fragmentation or climate change—can create new opportunities for pathogens that were previously circulating only in wildlife or environmental reservoirs to spill over into people or livestock hosts [1]. The ecological drivers of pathogen spillover have become a focus of attention after a series of high-profile spillover events, including avian influenza, Ebola and Hendra viruses. Spillover to humans can be common for some disease agents, as in the case of Lyme disease, where every human case is a spillover event from a wildlife reservoir; or rare, as with HIV, which emerged after a handful of spillover events of simian immunodeficiency virus mutated into HIV [2]. While it would be ideal to prevent spillover, especially in cases like Ebola virus and HIV, where onward transmission leads to many human cases, data on the best way to mitigate risk at specific points along the spillover process are lacking.

Here, we focus on ecological interventions: actions that target the ecological context in which the spillover process occurs. We distinguish between ecological interventions and conventional interventions. We make this distinction as a practical way to focus our attention on novel (ecological) interventions and distinguish them from more conventional approaches in the medical and veterinary literature that have been well-treated previously, although we acknowledge that the designation of ‘ecological’ versus ‘conventional’ can be context-specific and not mutually exclusive. We define conventional interventions as medical and veterinary approaches, like disinfection, vaccination and treatment, that have been used widely by public health communities and focus primarily on the medical or chemical management of risk in human or domestic animals, or their immediate environments, without regard to more complex ecological interactions. While acknowledging successful conventional interventions, here, we focus on systems-based approaches that target spillover by harnessing a better understanding of a system's ecology. For example, although culling the reservoir and mass vaccinating the spillover hosts indeed change the ecology of pathogen transmission, here we expand to a diverse set of additional interventions that target the natural interactions or ecosystem services that occur upstream or downstream in the spillover process. If we can better understand the disease ecology, including the interactions among disease-carrying organisms, or between organisms and their complex environments contributing to spillover, we may be able to devise novel, actionable solutions to manage or reduce spillover (for example, augmentation of natural enemies, habitat modification or restoration of ecosystem services such as water purification provided by wetlands, etc.; table 1). Drawing on real-world examples of well-studied spillover systems, we outline some important collective insights and general concepts about successfully using ecological interventions to manage spillover.

**Table 1.** Spillover barriers and associated conventional and ecological interventions that target each barrier layer.

location	spillover barrier	conventional intervention	ecological intervention	examples of ecological interventions	status	intervention no. (figure)
zoonotic reservoir	reservoir density or distribution	fences, culling	habitat modification	altered food distribution on elk feeding grounds to reduce brucellosis [3].	demonstrated, with correlational/observational support	1
			natural enemies	maintenance of leopard populations to limit rabid feral dog populations [4]. See also [5].	hypothesized	2
	pathogen prevalence (in reservoir)	chemotherapy, vaccination of reservoir, test and remove	dilution hosts	increased diversity of host community for *Ixodes* ticks (e.g. by increasing size of forest fragments) may increase abundance of incompetent hosts for *Borrelia burgdorferi*, reducing Lyme disease spillover [6].	demonstrated, but generality of dilution effect of increased biodiversity is debated [7–9]	3
			genetic management	reducing population size and stay-time of poultry in markets minimizes prevalence and genome reassortment of influenza viruses [10].	demonstrated	4
	infection intensity or pathogen shedding		reservoir nutrition and susceptibility	supplementing key flowering tree food resources for flying foxes (via habitat conservation/restoration) to boost nutrition and immunity in bats and decrease viral shedding rates of Hendra by bats [11], or similarly preserving native prey communities for vampire bats (rabies) via habitat conservation/restoration, which also encourages bats to feed on wildlife rather than humans or livestock. See also [12].	hypothesized	5
environment	pathogen survival and spread	insecticides, disinfection	habitat modification	*Anopheles* (malaria) [13] and *Culex* (West Nile virus, Japanese encephalitis, St Louis encephalitis, also filariasis) mosquito reductions by fish additions to rice fields, while simultaneously increasing rice yields [14].	hypothesized	6
			gene management	gene drive in *Anopheles gambiae* to control spread of *Plasmodium* spp. causing malaria [15].	demonstrated	7
			natural enemies	maintaining the scavenger community (e.g. eagles and coyotes in the USA, vultures in India [16]) as an important consumer of carcasses that harbour Brucella, anthrax, and other pathogens. See also [17].	demonstrated, with correlational/observational support	8
spillover host	spillover host exposure	chemical repellents, biosecurity	human behaviour modification	bamboo skirts over date palm sap collection pots to reduce bat contamination of sap with Nipah virus in Bangladesh [18,19]. See also [20].	demonstrated	9
	spillover host susceptibility and infection	chemotherapy, vaccination	managing coinfections or microbiome, genetic management	the use of faecal transplant procedures to treat *Clostridium difficile* with microbial competitors [21].	demonstrated	no corresponding number in figure

## 2. Ecological interventions targeting different spillover barriers

**Figure 1.**
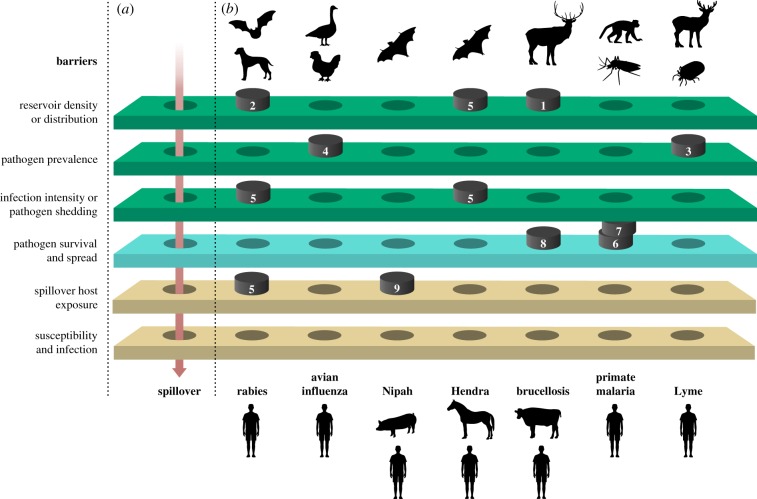
Ecological interventions to manage spillover. Ecological interventions may offer creative solutions to reduce or prevent spillover at various barrier layers of the spillover process. The barriers occur in reservoir hosts (green), the environment and vectors (cyan) and spillover hosts (beige). For spillover to occur, holes in the barriers need to line up in space and time (*a*). To prevent this, interventions can be applied to reduce the sizes of the holes, or prevent the holes from aligning in space and time. The black numbered plugs blocking the holes represent some example ecological interventions (numbers refer to interventions in table 1) that could be implemented to manage spillover (*b*).

The ecological processes governing spillover can be described as a series of barriers that a pathogen must overcome to eventually traverse from the vertebrate reservoir to the final spillover host at a particular place and moment in time [22]. While our understanding of disease ecology is improving, options to manage or control spillover in wildlife hosts remain limited. Conventional solutions like culling, vaccination and chemical control (e.g. drugs, insecticides and disinfectants) can have adverse consequences such as environmental damage, the evolution of resistance or non-target effects, and are often logistically challenging to implement. In general, we can prevent or limit spillover by reducing or preventing the flow of pathogens across one or more of the potential barriers (e.g. managing population size or prevalence in reservoir hosts, pathogen persistence in the environment, or vector abundance, or by changing reservoir distribution or contact between reservoir and spillover hosts to prevent the pathogens and hosts from aligning in space and time). An ecological intervention may also target pathogen flow in several layers and systems, not just one (figure 1).

A major difference between many ecological and conventional interventions is in how these actions alter the pathogen transmission process. Many conventional management actions directly—and often temporarily—change the numbers of susceptible, infectious and recovered individuals. This is true, for instance, of vaccination (which reduces the number of susceptible individuals), culling (which temporarily reduces reservoir host density) and test-and-slaughter (which temporarily reduces diseased individuals but also reduces herd immunity). However, without sustained management effort, the effects of these actions can wane. This was observed in human measles, for instance, whereby measles risk increased following vaccination disruption after the 2014 Ebola epidemic in Sierra Leone, Liberia and Guinea [23]; we would expect the same pattern to emerge in many vaccination scenarios aimed at managing spillover. Ecological interventions, on the other hand, try to manage the underlying transmission processes, based on ecological understanding. For instance, introduction or restoration of a natural enemy through conservation of its habitat could impose a longer-term change on host mortality rates than a single reduction in host density owing to culling (table 1 and figure 1) and increasing host genetic diversity might provide a lasting reduction in susceptibility [24].

Here, we begin to explore some of the complexities involved in designing effective solutions that target ecological processes involved in zoonotic spillover. We focus on case studies that demonstrate logical ecological interventions that can (or have been proposed to) control the density, distribution or infectiousness of vertebrate reservoir hosts; survival or spread of pathogens in the environment; or contact risk, susceptibility or treatment success in the focal spillover host (table 1 and figure 1).

### (a) Targeting reservoir hosts: moving beyond culling towards alternative non-lethal approaches

Throughout history, culling the reservoir host has been a common intervention for reducing spillover risk from wild or domestic vertebrates, but culling often incurs unacceptable economic or ecological costs, or unintended negative consequences [25,26], such as potential increases in pathogen transmission or virulence [25,27]. For example, Nipah virus was first discovered after it caused encephalitis outbreaks in Malaysia and Singapore among people involved in raising or slaughtering commercial pigs [28]. Culling pigs was effective at managing disease risk for people; however, there was substantial economic fallout, including production losses and the loss of approximately 36 000 jobs from farms that were not re-opened after the pigs were culled [29] (table 1). It was soon discovered that the natural reservoirs of the virus included several species of flying fox (i.e. *Pteropus* spp. bats). An ecological intervention to reduce transmission from bats to pigs was devised as a more sustainable solution to manage spillover: policies were put in place that required fruit trees, which attract bats and were implicated as the pathway for multiple spillover events on the outbreak's index farm, to be planted a minimum distance from pig sties [12] (table 1). Because the pig farming communities were heavily affected by the outbreak and incurred minimal cost from adopting this practice, this relatively simple ecological intervention has prevented further outbreaks of Nipah virus in Malaysia since 1998 [12].

Rabies control has also relied on culling at the level of the bat reservoir host. In Latin America, bats account for more cases of rabies than canines or other carnivores [30,31], and control efforts focus on the main reservoir host, the common vampire bat (*Desmodus rotundus*) [32,33]. Control efforts have included destruction of roosts, which indiscriminately kill other bat species in addition to vampire bats [34], alongside the application of a lethal anticoagulant paste applied to captured bats that spreads through colonies via allogrooming at the roost [33,35]. Recent studies suggest that rabies seroprevalence in vampire bats was highest in bat colonies with a history of culling, and that culling might inadvertently increase viral transmission by altering vampire bat movement [25,36].

Culling can alter host movement dynamics, in tandem with host densities, leading to unexpected disease consequences, as shown in several well-studied systems (e.g. *Mycobacterium bovis* in badgers; *Mycoplasma aggasizi* in desert tortoises). Culling can also alter pathogen dynamics in the reservoir host through increased population turnover. For instance, a theoretical analysis of classical swine fever in wild boars showed that culling led to the counterintuitive result that both disease prevalence and absolute number of infectious individuals increased as a consequence of host population reduction [27,37]. Studies of test-and-cull in bison and elk in an effort to control brucellosis have produced similar counterintuitive results, whereby herd immunity was reduced, resulting in subsequent outbreaks [38]. Thus, (often reactive) culling practices can be an effective intervention in controlling wildlife diseases, or can be ineffective, especially where efforts are not spatially coordinated and do not account for important nonlinearities and heterogeneities in disease transmission and host demography.

Beyond culling, there are many non-lethal interventions that can be employed to reduce spillover at the level of the reservoir host, including reservoir-host vaccination, treating infections or co-infections and ecological interventions such as contact or connectivity manipulations (e.g. fences and translocation), or fertility control. Oral vaccination of vampire bats has been proposed to reduce rabies spillover by capitalizing on the same social behaviour that facilitates anticoagulant-based bat culling efforts (table 1). Yet, while vaccination of reservoir hosts has been a successful alternative to culling for terrestrial rabies control in North America and Europe, no commercial vaccine is available for rabies control in vampire bats [39].

Complications associated with widespread vaccination campaigns are not limited to rabies. For many wildlife pathogens, vaccines are unavailable, costly to develop and deploy, and logistically challenging to implement at appropriate spatial and temporal scales [40,41]. Even where vaccines for reservoir hosts are available, vaccination is sometimes not socially acceptable. For example, after the recent development of a highly effective Hendra virus vaccine for horses, social factors including spread of anti-vaccination information by some members of the community, cost of the vaccine and export implications for vaccinated horses has meant that vaccine uptake is relatively low [42]. Similarly, in the case of avian influenza, decreasing spillover risk at the wild bird–poultry interface through vaccination may not always be effective against newly (rapidly) evolving strains, and vaccination of poultry is sometimes not affordable owing to the large number and high turnover of poultry, relative to the rare frequency of spillover of highly pathogenic avian influenza strains [43].

Employing natural enemies to control disease may sometimes be more effective and less costly than culling and can have additional benefits for the environment, like restoring threatened or endangered predators [44]. Predators are likely to affect the diseases of their prey through several mechanisms: killing sick individuals [43,44], lowering prey population size and altering aggregation patterns. For these reasons, wolf management has been proposed as a potential intervention for reducing chronic wasting disease and brucellosis in elk [5,45], but this ecological intervention has not been fully implemented owing to potential societal costs associated with larger wolf populations.

Ecological interventions to control reservoir host movement, connectivity or distribution have sometimes been employed, with variable success owing to opposing impacts on multiple layers of the spillover process. For example, food distribution to keep elk away from cattle during winter months and to reduce risk of brucellosis spillover has been ongoing for many decades in the Yellowstone area [3]. However, while supplemental feeding helps to separate elk and cattle, it also concentrates elk on feed grounds during winter, potentially elevating brucellosis prevalence within the wildlife reservoir and increasing the spillover risk associated with contacts that do occur [46,47]. Research continues to flesh out the multiple interacting effects of supplemental feeding, but to date, the effects are equivocal [48,49] and it is hard to detect any benefit in this highly variable system with many environmental drivers.

Ultimately, ecological approaches targeting the reservoir require a sophisticated understanding of the structure of, and processes involved with, the various components of the reservoir community [50]. Gaps in our understanding of the complex ecology of reservoirs have hindered progress in managing spillover of Ebola virus and rabies virus [51,52], among other zoonotic pathogens. Interventions can offer important clues to disentangle which reservoir components are most important to spillover [53]. For example, in Zimbabwe, sylvatic canids may play a role in the maintenance and spillover of human rabies in some areas. If domestic dogs are the main reservoir and source of spillover cases in people, then a campaign vaccinating domestic dogs within a region should lead to strong reductions in human infection, but if jackals are a secondary component of spillover risk (which some studies suggest) then oral baiting of jackals with rabies vaccine may be additionally required to reduce human rabies [50]. This illustrates a broader theme in spillover management, namely, that one strategy does not fit all cases owing to differences in reservoir ecologies.

### (b) Targeting the environment: habitat, vector control and ecosystem management

Understanding pathogen persistence in abiotic environmental reservoirs sometimes leads to simple interventions that operate on many interacting levels to manage spillover risk. For example, spillover transmission of avian influenza can be managed in live-bird market systems by ‘rest days’ (during which no birds are brought to market) and lessening stay-time in markets; if birds are removed before the virus can infect and become infectious in a new host, then outbreaks can be avoided [54]. Limiting stay-time also serves to strongly reduce viral genome reassortment (gene shuffling that can result in novel strains that may have expanded host range or higher virulence in donor hosts) of avian influenza in retail markets by limiting co-infection and thus reducing the probability of generating novel spillover strains [10]. For Hendra virus spillover, blocking horses' overnight access to trees in pastures has been proposed as a solution to prevent viral transmission from bats to horses, since this intervention would delay horses’ access to grass contaminated by bat urine (if bats happen to roost in those trees), thereby reducing the probability that a horse would come into contact with recently secreted, live Hendra virus [55].

Targeting the environmental components in the ecology of disease transmission has a long history in vector management. For example, vector control using chemical pesticides has been a primary method of defence in reducing vector-borne disease risk, but this conventional intervention is prone to limitations, such as resistance evolution, non-target effects and environmental damage [56]. Chemical control can be replaced or enhanced by stocking mosquito predators in mosquito breeding habitats and this strategy has been used in diverse habitats to control disease-carrying mosquito vectors, including ponds, cisterns, irrigation canals and rice fields, with mixed success [57,58]. Similarly, control of blacklegged ticks (*Ixodes scapularis*, a Lyme disease vector) by spraying entomopathogenic fungi (e.g. *Beauveria bassiana* or *Metarhizium anisopliae*) on pastures has shown promise [17].

Natural habitat manipulation to reduce environmental persistence of pathogens has been used less, but holds promise. For example, scavengers like vultures compete with spillover pathogens for host tissue (a form of intra-guild predation). In India and Pakistan, declines in vulture populations owing to lethal effects of an anti-inflammatory drug, diclofenac, have resulted in increased volumes of uneaten carcasses, which act as environmental breeding grounds for diverse zoonotic spillover pathogens including anthrax, brucellosis and bovine tuberculosis [59–61]. Feral dog populations have also grown owing to increased access to carcasses, and although a causal association has not been definitively established, correlative evidence suggests that loss of vultures indirectly led to an increase in dogs and human rabies spillover [62]. Recent policy reform in India and Pakistan, banning diclofenac, may allow wild vulture restoration and lead to both conservation and public health benefits.

### (c) Targeting the interface between reservoir and spillover hosts

Spillover can increase when landscape modification—like habitat encroachment, agricultural expansion and road building—increase contact rates between reservoir and spillover hosts [63]. Targeting this interface can sometimes offer the most effective interventions for reducing spillover, but interface controls could operate at a variety of scales. For instance, the use of bed nets to curb malaria is a classic example of controlling the interface between mosquitos and people. In addition, a combination of ecological and conventional interventions have helped reduce Hendra virus spillover risk in Australia by preventing contact at the interface between horses and flying fox urine (e.g. covering food and water, keeping horses away from fruiting and flowering trees [55]) and preventing exposure at the horse–human interface through use of personal protective equipment for veterinarians and owners dealing with sick horses [20].

Biosecurity is another example of a conventional intervention to reduce spillover along the wildlife–domestic animal interface. For example, biosecurity efforts to reduce rates of contact appropriate for avian influenza virus transmission between wild birds and poultry have been an important component of avian influenza risk management. But identifying biosecurity measures that prevent exposure can be challenging [64]: prior to 2014, no highly pathogenic avian influenza had been detected in the USA but then, after three different highly pathogenic reassortants were detected almost simultaneously in wild birds, these strains soon caused at least 18 independent emergence events in US commercial poultry operations, despite biosecurity measures [64,65].

Ecological interventions aimed at the interface between donor and recipient hosts have sometimes targeted shared food resources [48]. For example, Nipah virus in Bangladesh can be transmitted to people through drinking uncooked date palm sap contaminated by excreta from infected fruit bats [66,67]. By limiting bat access to sap that is drip-collected in clay pots overnight, viral contamination by bats can be reduced [19,68]. In principle, this should be an effective and acceptable ecological intervention because it only needs to be implemented on trees from which sap will be collected for drinking. However, wholesale adoption of this approach, relying on modifications to human behaviour, has been difficult to achieve across Bangladesh [18,69].

There can be ecological interventions that act at the scale of habitat modification to alter the contact rate of reservoir and spillover hosts. For example, forest fragmentation, wildlife population declines and the proliferation of cattle rearing have prompted shifts in vampire bat feeding from wildlife to human and livestock prey [70]. It has been proposed that rabies vaccination of livestock might be a viable conventional intervention [71]. Yet, if vaccination coverage is low, and livestock density continues to increase, then growing bat populations reliant on cattle near human settlements might still worsen rabies spillover risk to humans, despite a livestock vaccine [72,73]. Also, because vampire bats preferentially feed on livestock, even when wildlife are available [74], rapid withdrawal of livestock has been associated with prey switching to humans by vampire bats, with consequent increases in human rabies [75,76]. There might be more durable, conservation-based approaches to mitigate bat–human contact, or reduce forest-to-agricultural edge habitat where bats are exposed to cattle, but more research about how shifting prey distributions could impact vampire bat feeding ecology is needed to disentangle many complex and interacting factors [71].

Similarly, the movement ecology of traditionally nomadic flying foxes (*Pteropus* spp.) in Australia has shifted owing to the loss of critical nectar resources after land clearing for agriculture and urban development [11]. Flying foxes, in turn, experience acute episodes of nutritional stress [77]. To decrease the energetic costs of foraging, colonies split into many smaller populations that remain close to consistent but poor-quality urban food resources [78]. Nutritional stress and urban habituation likely drive shedding of Hendra virus from these reservoir hosts as well as more contact with equine recipient hosts [79]. One proposed habitat solution to this problem has been to restore native winter nectar habitat patches to draw flying foxes out of urban areas, away from horses and people and towards their preferred resource [79].

### (d) Targeting susceptibility and infection in spillover hosts

Conventional biomedical approaches remain an important tool in managing spillover and may be synergistic with ecological interventions applied at processes that are upstream in the spillover chain. Treating human or livestock cases, treating co-infections (to reduce susceptibility) and vaccinating recipient hosts are classic examples and remain necessary tools to preserve public health. But, particularly where treatments or vaccines are not available or not affordable, such as for understudied pathogens and in resource-poor settings, taking advantage of synergies with ecological approaches along the spillover hierarchy may be beneficial.

## 3. Modelling and measuring disease systems to find potential interventions to reduce spillover

Modelling a system to explore sensitivity to various interventions (as well as costs and benefits) can help determine which interventions are most important, which ones are not viable and which ones require more monitoring data for better decisions. Even simple model systems can demonstrate nonlinearities in outcomes that make straightforward comparisons of interventions difficult (box 1). Sometimes thresholds emerge that can be advantageous for control (e.g. cost-effective interventions that disproportionately reduce spillover in the recipient host with relatively little effort). Other times, nonlinearities introduce challenges when an intervention results in a large reduction in a particular parameter (or set of parameters) but may have little effect on spillover rates to recipient populations if a particular disease-transmission threshold is not surpassed. For example, box 1*c* shows a threshold effect in a simple simulation of a hypothetical spillover disease system (parameterized to resemble bat–human spillover of viruses with high human-to-human transmission, like Ebola): treatment of the donor (bats) almost eliminates disease in the recipient when the coverage is greater than 99%, but has little to no effect at lower intervention intensities. Our toy model also illustrates that understanding the dynamics in both host species is critical because some interventions could inadvertently increase spillover rates to recipient hosts (i.e. negative ecological feedback). For example, in box 1*c*, representing a disease with high human-to-human onward transmission after spillover (like Ebola), behaviour modification of the recipient (humans) to reduce contact with other sick people and rapid treatment of human cases were the most two most sensitive interventions in reducing total human disease; however, these measures also resulted in an increase in the total number of *spillover* transmissions (even while reducing the total number of human cases; see electronic supplementary material) because of a consequent build-up of susceptible people in the system. A simulation approach like the one we present here can enable visualization of complex outcomes, including unintended consequences, which could be useful for designing formal tests of ecological interventions for managing spillover. The exercise we present here is intended to be illustrative, not prescriptive, because tailoring models of this sort to specific systems in order to guide real management decisions would require a better parameterization effort, including deep understanding of the ecological dynamics of donor and recipient hosts and the potential for density-dependent processes (e.g. the possibility of compensatory population growth in response to culling activities); see, for example, [80] in this issue.

Box 1. **A simple model system simulating stochastic Susceptible–Infectious–Recovered disease dynamics, involving transmission among donor (reservoir) and recipient (focal) hosts, coupled by spillover. (*a*) Model schematic (for details, see electronic supplementary material). (*b*,*c*) Heat maps simulating cumulative cases in the recipient, given a set of interventions applied to varying degrees (ecological and conventional interventions targeting different model parameters). An ‘intervention intensity’ of 0 represents the base case scenario, with no intervention, and all other intervention intensities can be compared to the base case in each column. This exercise demonstrates the nonlinearities that emerge when comparing potential interventions in a relatively simple, but qualitatively flexible, spillover system. This model is flexible enough to qualitatively represent several types of spillover diseases, including those where onward transmission from human to human is limited (as in *b*), like (but not parameterized exactly as) Nipah virus, and those where onward transmission is high (as in *c*), like (but not parameterized exactly as) Ebola virus. See electronic supplementary material for more details on model structure, parameterization and results of the simulations.**

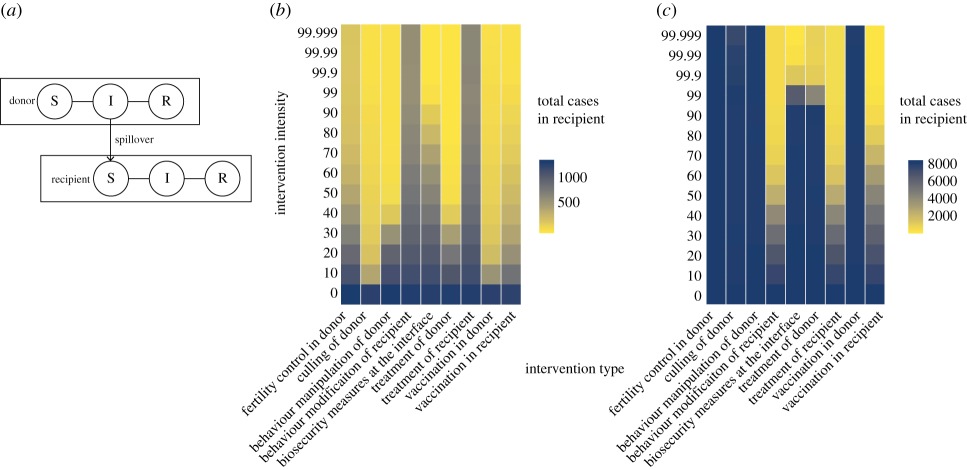

## 4. Economic, social and political considerations can determine success or failure in managing spillover

How can we integrate ecology, public health, stakeholder perspectives and economics into the recommendations for managing ecological interventions to reduce spillover risk? It is not straightforward for a manager to decide which intervention to invest in, and whether it should be ecological, conventional, or both. In general, successful implementation of an ecological intervention requires: knowledge (we must be aware of and understand the intervention), means (both financial and logistical), mandate (jurisdiction) and motivation (benefits outweigh costs and those incurring costs also realize the value of the benefits) [81]. When the benefits of an action (e.g. reduced spillover) do not align with where (and by whom) the costs are incurred (e.g. one particular sector), social and political attention to aligning or subsidizing those costs and benefits across sectors may be necessary, and this is difficult.

In particular, ecological interventions that target habitats and natural populations are likely to fall under the jurisdiction of government agencies that have mandates other than human or livestock health. So, wildlife and land management agencies may have the means and mandate, but do not necessarily have the motivation. Not all ecological interventions will be win–win for all interested parties. In some cases, reducing wildlife densities or manipulating habitats to improve human or livestock health may not be a priority for hunter or conservationist communities. In this case, more collaboration among sectors and the sharing of costs and benefits will be essential, and yet difficult to implement.

Just as for new biomedical tools (e.g. drugs or vaccines), new potential ecological interventions should not be rolled out wholesale, everywhere, until their safety and effectiveness have been evaluated. Or, if this is not possible owing to the urgency of a situation, interventions could be implemented in an adaptive management framework, with attention to monitoring both the effectiveness of the intervention and comparable controls, wherever possible [82].

Conversely, sometimes potentially effective tools still fail because of social, economic or political constraints. For example, decreasing wolf hunts and removals has not been implemented as an intervention to reduce brucellosis, owing to the potential predation risk to livestock as well as the interests of some in the hunting community to maintain large populations of elk. Also, the anti-vaccination movement highlights how even conventional interventions like vaccination, although relatively safe and effective, are not without controversy.

## 5. Conclusion

Spillover involves cross-species pathogen transmission across a highly complex landscape of ecological processes, which calls for ecological solutions. In this piece, we introduce the notion of an ecological intervention as a potentially underused approach to find effective, long-lasting and creative solutions to reduce spillover, with minimal environmental damage. Moreover, ecological interventions can be complementary, not antagonistic, to conventional approaches, which often target different barriers in the spillover process. However, conventional interventions such as culling and medical treatment are often reactive, short-lived, and can introduce further complications: culling can sometimes inadvertently enhance disease transmission, drugs can enhance virulence and/or alter resistance, and for many spillover diseases, vaccines and effective treatments are not yet available. In these cases, managing upstream risks using ecological interventions may be the best option. Ecological interventions, like many conventional ones, are not without their caveats and controversies. Social, political and economic considerations can limit broad changes to ecosystems that are sometimes needed to implement ecological interventions. Here, we have explored some of the next steps towards identifying and implementing effective interventions to manage or reduce spillover. Examples of ecological interventions provided here target reservoir hosts (i.e. preventing wildlife–livestock contact), the environment (i.e. ecosystem management) and the whole spectrum of the interface between ecological reservoirs and people (or other focal hosts like livestock). Finally, we demonstrate a simple modelling framework for visualizing the complex and nonlinear effects of various interventions for simple disease spillover systems. By better understanding and harnessing our understanding of complex ecological systems, ecological interventions might offer new ways to design cost-effective, socially acceptable, sustainable interventions that can reduce spillover risk.

## Supplementary Material

Modelling methods

## Data accessibility

This article has no additional data.
