# Supplementary material for: Ecological interventions to prevent and manage zoonotic pathogen spillover
Source: Philos Trans R Soc Lond B Biol Sci. 2019 Aug 12;374(1782):20180342. doi: 10.1098/rstb.2018.0342 (PMC6711299; doi:10.1098/rstb.2018.0342)
Supplement: Modelling methods [file rstb20180342supp1.pdf]

Ecological interventions to prevent and manage pathogen spillover - Supplemental Information

Sokolow, SH, N Nova, K Pepin, AJ Peel, K Manlove, PC Cross, DJ Becker, RK Plowright, JRC Pulliam, H McCallum, and GA De Leo

April 30, 2019

Modelling methods

We defined a simple 2-host system, i.e., donors and recipients, with three compartmental classes, Susceptible-Infected-Recovered (SIR), for each host species, and spillover from donors to recipients [1]. Model simulations are conducted using an Euler-multinomial approximation to the two-host ordinary differential equation model. Our model builds off previously proposed frameworks (e.g. [2, 3]), but our focus is more on the practical implications of potential interventions. Using a tractable framework, we focus on the comparative outcomes of simulated management options applied to either donor or recipient populations and highlight potential non-linearities in spillover risk that result.

We use the model to simulate disease dynamics for two sets of fixed parameter values (Table 1). We examine how each particular ecological intervention applied to a single parameter (process) affects disease outcomes in recipient populations in terms of: 1) the total number of cases in the recipient population, and 2) the total number of spillover events in a defined timeframe.

Interventions were implemented as a fixed proportional reduction in a parameter value, except culling and vaccination, which were specified as annual proportions that were then converted to rates. We assumed that each specified intervention affected a single parameter.

Each simulation was run for 5 years using daily time steps with initial population sizes of 10000 hosts in each of the donor and recipient populations. We initialized all simulations at the endemic equilibrium (with values rounded) for the donor and at the disease-free equilibrium for the recipeint. We used parameter values representing two different example spillover systems, which differed in their assumed contact rates and durations of infection. We ran 1000 replicate simulations per ecological intervention condition. We present the average outcomes (total cases in the recipient population and total number of spillover events) in Figures S3-S6.

Model specification and assumptions are described below. Note that the modeling framework is intentionally simplistic because our goal is to visualize potential non-linearities in effects of different ecological interventions. This type of framework could be adapted to address ecological complexities of particular systems (e.g., environmental transmission, spatial structure, etc.). Currently, the framework is intended to generate hypotheses for further examination.

Assumptions

- Only spillover from donor to recipient, no spillback from recipient to donor
- Homogenous mixing in each population and between them
- Direct contact transmission only
- Lifelong immunity from infection or vaccination
- No disease-induced mortality
- All newborns are susceptible
- No spatial structure
- Density-dependent transmission; no other density-dependent processes
- Occaisional re-introduction of the pathogen into the donor host to prevent extinction (as would be expected if the pathogen were maintained in a donor host via metapopulation dynamics)

Model structure

We first specify the ordinary differential equation (ODE) model, based on the classic SIR compartmental framework (e.g., Keeling and Rohani 2007). We then implement a stochastic, discrete time approximation to the ODE system using an Euler-multinomial approach. Disease dynamics in the donor host (d) are described by the following equations:

$$\frac{dS_d}{dt} = \mu_d N_d - (\delta_d + \alpha_d)S_d - (\beta_{d-d} I_d + \rho_d)S_d - \nu_d S_d$$

$$\frac{dI_d}{dt} = (\beta_{d-d} I_d + \rho_d)S_d - (\delta_d + \alpha_d + \gamma_d)I_d$$

$$\frac{dR_d}{dt} = \gamma_d I_d + \nu_d S_d - (\delta_d + \alpha_d)R_d$$

and in the recipient host (rr):

$$\frac{dS_r}{dt} = \mu_r N_r - \delta_r S_r - (\beta_{r-r} I_r + \beta_{d-r} I_d)S_r - \nu_r S_r$$

$$\frac{dI_r}{dt} = (\beta_{r-r} I_r + \beta_{d-r} I_d)S_r - (\delta_r + \gamma_r)I_r$$

$$\frac{dR_r}{dt} = \gamma_r I_r + \nu_r S_r - \delta_r R_r$$

$$\frac{dS_r}{dt} = \mu_r N_r - \delta_r S_r - (\beta_{r-r} I_r + \beta_{d-r} I_d)S_r - \nu_r S_r$$

$$\frac{dI_r}{dt} = (\beta_{r-r} I_r + \beta_{d-r} I_d)S_r - (\delta_r + \gamma_r)I_r$$

$$\frac{dR_r}{dt} = \gamma_r I_r + \nu_r S_r - \delta_r R_r$$

where all parameters are defined as in Table S1. For simulations wtih vaccination ($\nu_d > 0$$\nu_d > 0$ or $\nu_r > 0$$\nu_r > 0$), the vaccination hazards (daily rates) are calculated from average fraction of hosts in the population vaccinated every year $0 < p_i < 1$$0 < p_i < 1$ as $\nu_i = -ln(1 - p_i)/\Delta t$$\nu_i = -ln(1 - p_i)/\Delta t$, where $\Delta t = 365.25$$\Delta t = 365.25$. For simulations wtih culling ($\alpha_d > 0$$\alpha_d > 0$), the excess mortality hazard (daily rate) is similarly calculated from the annual fraction culled.

For this system, we can derive reproduction ratios R_0 R_0 for sub-component models (assuming no vaccination, i.e., $\nu_d = \nu_r = 0$ $\nu_d = \nu_r = 0$), namely:

$$R_{0_{d-d}} = \frac{\beta_{d-d} N_{0_d}}{\delta_d + \gamma_d}$$
$$R_{0_{d-r}} = \frac{\beta_{d-r} N_{0_r}}{\delta_d + \gamma_d}$$
$$R_{0_{r-r}} = \frac{\beta_{r-r} N_{0_r}}{\delta_r + \gamma_r}$$

$$R_{0_{d-d}} = \frac{\beta_{d-d} N_{0_d}}{\delta_d + \gamma_d}$$
$$R_{0_{d-r}} = \frac{\beta_{d-r} N_{0_r}}{\delta_d + \gamma_d}$$
$$R_{0_{r-r}} = \frac{\beta_{r-r} N_{0_r}}{\delta_r + \gamma_r}$$

Rearranging these equations allows the calculation of a transmission coefficient from the associated reproduction ratio: $\beta_{i-j} = R_{0_{i-j}}(\delta_i + \gamma_i)/N_{0_j}$ $\beta_{i-j} = R_{0_{i-j}}(\delta_i + \gamma_i)/N_{0_j}$.

Initial conditions are set based on the endemic equilibrium of the deterministic model (in the absence of interventions) for the donor and the disease free equilibrium for the recipient:

$$S_{0_d} = (\delta_d + \gamma_d)/(\beta_{d-d})$$
$$I_{0_d} = (\mu_d * N_{0_d}/S_{0_d} - \delta_d)/(\beta_{d-d})$$
$$R_{0_d} = \gamma_d I_{0_d}/\delta_d$$
$$S_{0_r} = N_{0_r}$$
$$I_{0_r} = 0$$
$$R_{0_r} = 0$$

$$S_{0_d} = (\delta_d + \gamma_d)/(\beta_{d-d})$$
$$I_{0_d} = (\mu_d * N_{0_d}/S_{0_d} - \delta_d)/(\beta_{d-d})$$
$$R_{0_d} = \gamma_d I_{0_d}/\delta_d$$
$$S_{0_r} = N_{0_r}$$
$$I_{0_r} = 0$$
$$R_{0_r} = 0$$

These values are rounded to the nearest integer for initiation of the Euler-multinomial approximation.

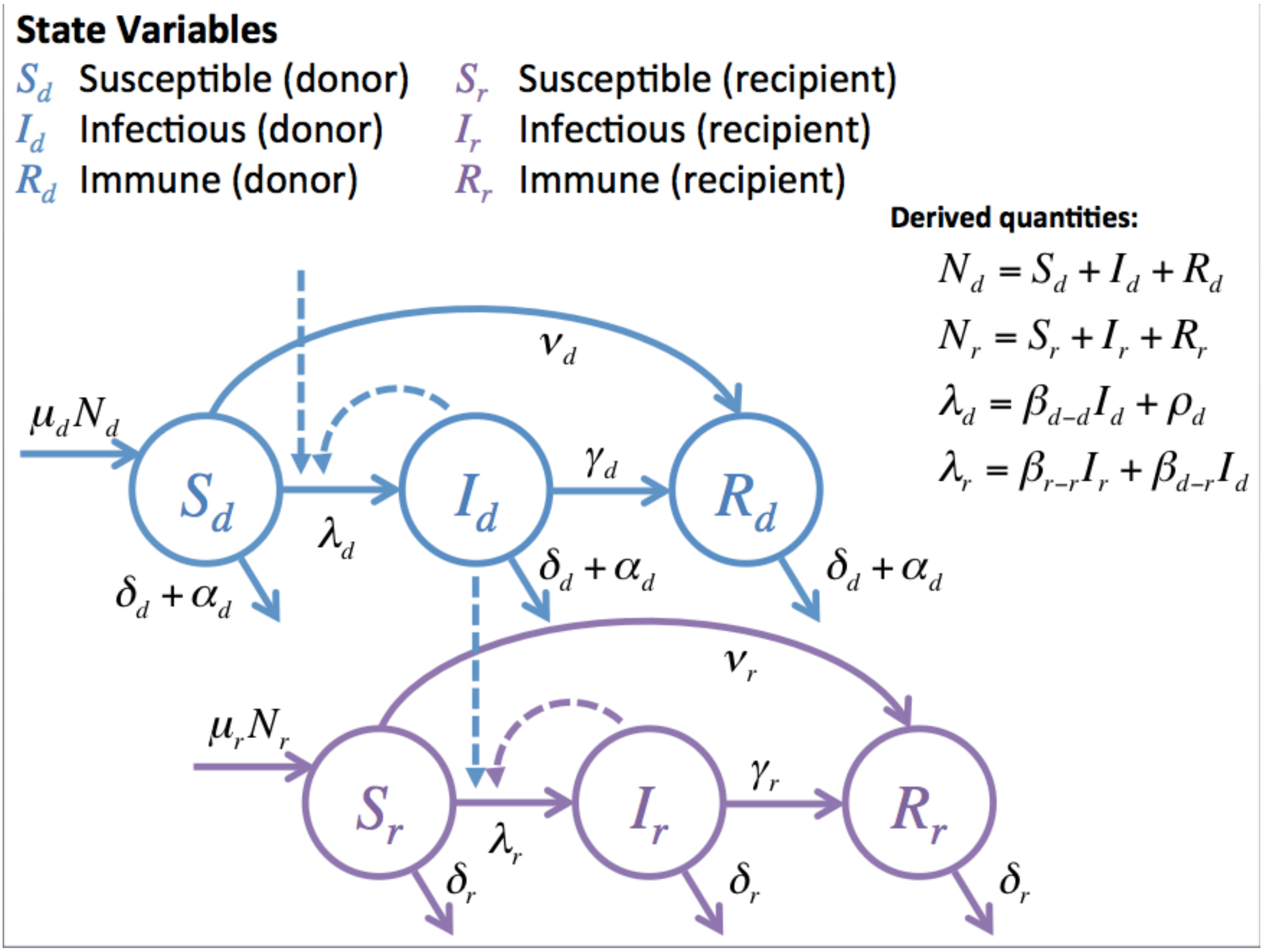

Figure S1: Structure of the two-host compartmental model used to explore the effects of ecological interventions. Solid arrows show rates of flow into and out of model compartments. Dashed arrows indicate influences that affect these rates.

Parameter definitions

Table S1 shows all parameter definitions, including both model notation and variable names used in the code for model implementation. Baseline parameter values are given for two example pathogens. Example 1 represents a pathogen that has supercritical transmission in the recipient host ($R_{0_{r-r}} > 1$ $R_{0_{r-r}} > 1$), like Ebola. Example 2 represents a pathogen that has subcritical transmission in the recipient host ($R_{0_{r-r}} < 1$ $R_{0_{r-r}} < 1$), like Nipah virus. In both examples, the donor and recipient life expectancies are set at 15 and 60 years, respectively, and birth rates are set to balance mortality rates ($\mu_i = \delta_i$ $\mu_i = \delta_i$).

Parameter	Description	Name	Units	Example 1	Example 2
N_{0_d} N_{0_d}	initial population size (donor)	pop0_d	individuals	10000	10000
N_{0_r} N_{0_r}	initial population size (recipient)	pop0_r	individuals	10000	10000
μ_d μ_d	per capita birth rate (donor)	birth_d	1/day	0.00018	0.00018
μ_r μ_r	per capita birth rate (recipient)	birth_r	1/day	4.6e-05	4.6e-05

δ_d δ_d	per capita mortality rate (donor)	mort_d	1/day	0.00018	0.00018
α_d α_d	per capita excess mortality hazard from culling (donor)	excess_d	1/day	0	0
δ_r δ_r	per capita mortality rate (recipient)	mort_r	1/day	4.6e-05	4.6e-05
ρ_d ρ_d	hazard of external infection (donor)	intro_d	1/day	1.4e-06	1.4e-06
β_{d-d} β_{d-d}	transmission coefficient (donor to donor)	beta_dd	1/day	1.9e-07	3.5e-05
β_{r-r} β_{r-r}	transmission coefficient (recipient to recipient)	beta_rr	1/day	1.2e-05	1.1e-05
β_{d-r} β_{d-r}	transmission coefficient (donor to recipient)	beta_dr	1/day	7.8e-08	2.3e-06
$R_{0_{d-d}}$ $R_{0_{d-d}}$	R_0 R_0 (donor to donor)	R0_dd	-	1.23	4.25
$R_{0_{d-r}}$ $R_{0_{d-r}}$	R_0 R_0 (donor to recipient)	R0_dr	-	0.5	0.28
$R_{0_{r-r}}$ $R_{0_{r-r}}$	R_0 R_0 (recipient to recipient)	R0_rr	-	1.9	0.55
$D_d = 1/\gamma_d$ $D_d = 1/\gamma_d$	duration of infection (donor)	dur_d	days	730	12
$D_r = 1/\gamma_r$ $D_r = 1/\gamma_r$	duration of infection (recipient)	dur_r	days	16	5
ν_d ν_d	per capita vaccination hazard (donor)	vax_d	1/day	0	0
ν_r ν_r	per capita vaccination hazard (recipient)	vax_r	1/day	0	0

Interventions

Table S2 shows how interventions relate to the model parameters. The `Type` column indicates how the intervention is referred to in the code. Intervention intensity is defined as a proportional scaling of the associated parameter, except for culling and vaccination (which have default values of zero). For culling and vaccination, the intensity is the average annual percentage of the relevant population that is culled or vaccinated.

**Intervention**	**Type**	**Description**
Fertility control in donor	fertCont_d	per capita birth rate (donor)
Culling of donor	cull_d	per capita excess mortality hazard from culling (donor)
Behavior manipulation of donor	reduceContact_d	transmission coefficient (donor to donor)
Behavior modificaiton of recipient	reduceContact_r	transmission coefficient (recipient to recipient)
Biosecurity measures at the	biosecurity	transmission coefficient (donor to recipient)

interface		
Treatment of donor	tx_d	duration of infection (donor)
Treatment of recipient	tx_r	duration of infection (recipient)
Vaccination in donor	vax_d	per capita vaccination hazard (donor)
Vaccination in recipient	vax_r	per capita vaccination hazard (recipient)

Model implementation

All code, simulation output, and other materials necessary to reproduce this file and results presented in the main text are provided at https://github.com/jrcpulliam/spilloverIntervention (https://github.com/jrcpulliam/spilloverIntervention). The Euler-multinomial approximation was implemented in R version 3.5.1 and uses the `pomp` package (version 1.19) [4,5]. A single update is accomplished using the following function:

```
# Single step for Euler-multinomial implementation of model
simEulerstep <- function (x, params, dt){
  with(c(as.list(x),params),{
    N_d <- S_d + I_d + R_d
    N_r <- S_r + I_r + R_r
    mort_d <- mort_d + excess_d
    dFOI <- beta_dd * I_d + intro_d # force of infection experienced by donor
    sFOI <- beta_dr * I_d # force of infection experienced by recipient from donor
    rFOI <- beta_rr * I_r # force of infection experienced by recipient from recip
ient

    births_d <- rpois(n=1,lambda=birth_d*N_d*dt)
    births_r <- rpois(n=1,lambda=birth_r*N_r*dt)
    S_d.removal <- reulermultinom(n=1,size=S_d,rate=c(mort_d,dFOI,vax_d),dt=dt)
    I_d.removal <- reulermultinom(n=1,size=I_d,rate=c(mort_d,1/dur_d),dt=dt)
    R_d.removal <- reulermultinom(n=1,size=R_d,rate=c(mort_d),dt=dt)
    S_r.removal <- reulermultinom(n=1,size=S_r,rate=c(mort_r,sFOI,rFOI,vax_r),dt=d
t)
    I_r.removal <- reulermultinom(n=1,size=I_r,rate=c(mort_r,1/dur_r),dt=dt)
    R_r.removal <- reulermultinom(n=1,size=R_r,rate=c(mort_r),dt=dt)

    # vector of changes
    c(
      dt, # change in time
      births_d-sum(S_d.removal), # change in S_d
      S_d.removal[2]-sum(I_d.removal), # change in I_d
      I_d.removal[2]-R_d.removal, # change in R_d
      births_r-sum(S_r.removal), # change in S_r
      S_r.removal[2]+S_r.removal[3]-sum(I_r.removal), # change in I_r
      I_r.removal[2]-R_r.removal, # change in R_r
      S_r.removal[2]+S_r.removal[3], # change in cum_I_r (total infections in reci
pient)
      S_r.removal[2] # change in cum_I_sp (total spillover infections)
    )
  })
}
```

A full realization is accomplished using the following function:

```
# Run a full realization for specified parameters and initial conditions
runSim <- function(init, pars, maxtime = round(YEARS*365.25), dt = TIMESTEP, brows
e = F){
  ts <- NULL
  pop <- init
  if(browse) browser()
  for(tt in seq(0,maxtime,dt)){
    ts <- rbind(ts,pop)
    pop <- pop + simEulerstep(pop,pars,dt)
  }
  return(data.frame(ts))
}
```

For simulations with interventions, the baseline parameter values are adjusted before they are passed to `runsim()`, as follows:

```
# Define parameter adjustments for interventions
intvPars <- function(prop,pars,intv = 'none'){
  switch(as.character(intv),
         none = {},
         fertCont_d = {
           pars['birth_d'] <- pars['birth_d']*prop # decrease birth rate
         },
         cull_d = {
           pars['excess_d'] <- toRate(1-prop) # convert annual proportion to daily
rate
         },
         reduceContact_d = {
           pars['beta_dd'] <- pars['beta_dd']*prop # behavior manipulation of dono
r
         },
         reduceContact_r = {
           pars['beta_rr'] <- pars['beta_rr']*prop # behavior modification of reci
pient
         },
         biosecurity = {
           pars['beta_dr'] <- pars['beta_dr']*prop # biosecurity measures at the i
nterface
         },
         vax_d = {
           pars['vax_d'] <- toRate((1-prop)) # convert annual proportion vaccinate
d to daily hazard
         },
         vax_r = {
           pars['vax_r'] <- toRate((1-prop)) # convert annual proportion vaccinate
d to daily hazard
         },
         tx_d = {
           pars['dur_d'] <- pars['dur_d']*prop # decrease duration of infection
         },
         tx_r = {
           pars['dur_r'] <- pars['dur_r']*prop # decrease duration of infection
         },
         error('Intervention unknown.'))
  return(pars)
}
```

Model output

Example trajectories

Example 1:

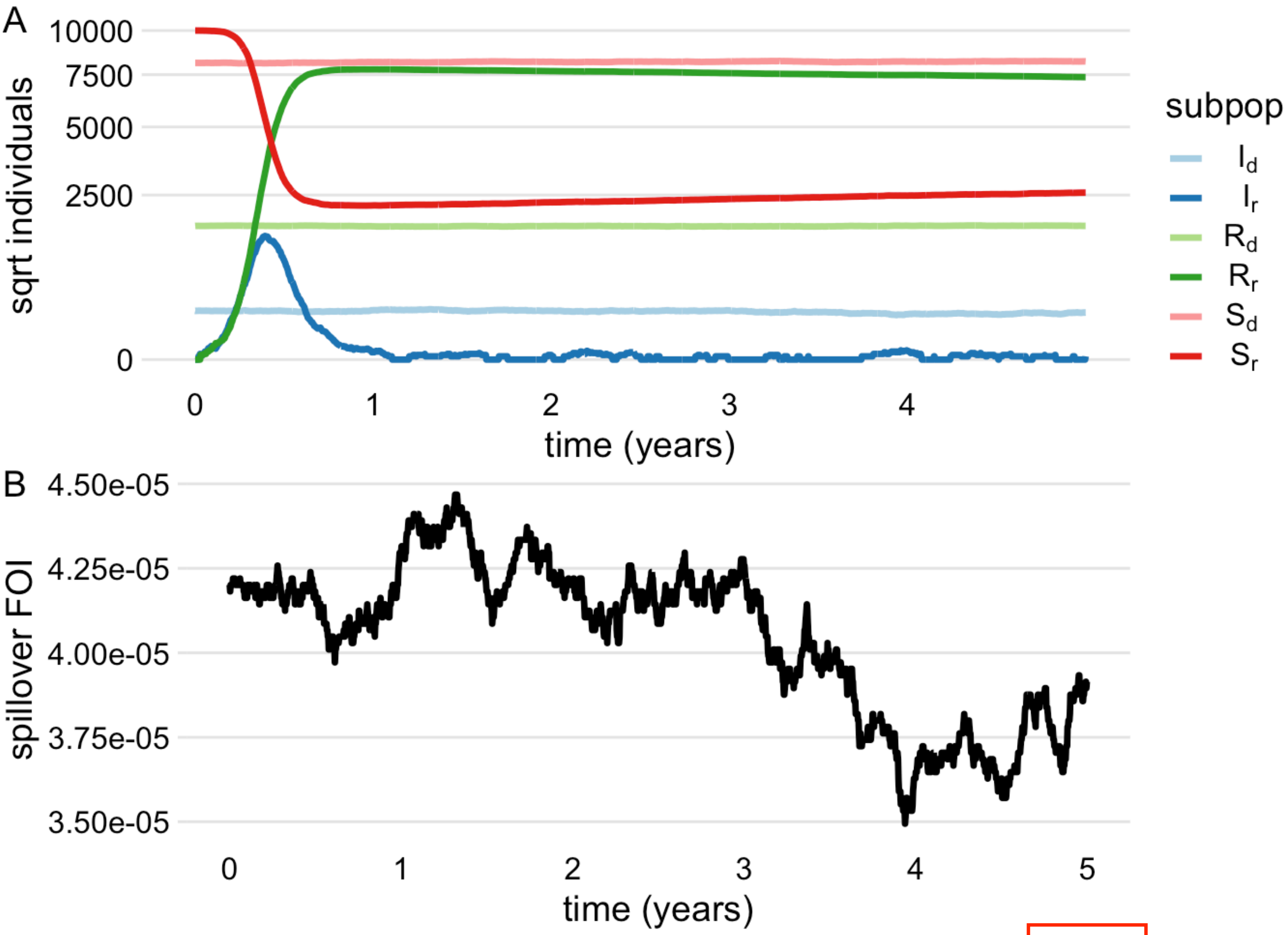

Figure S2: One random realization of dynamics for example system 1 ($R_{0_{r-r}} > 1$ $R_{0_{r-r}} > 1$), with no interventions.

Example 2:

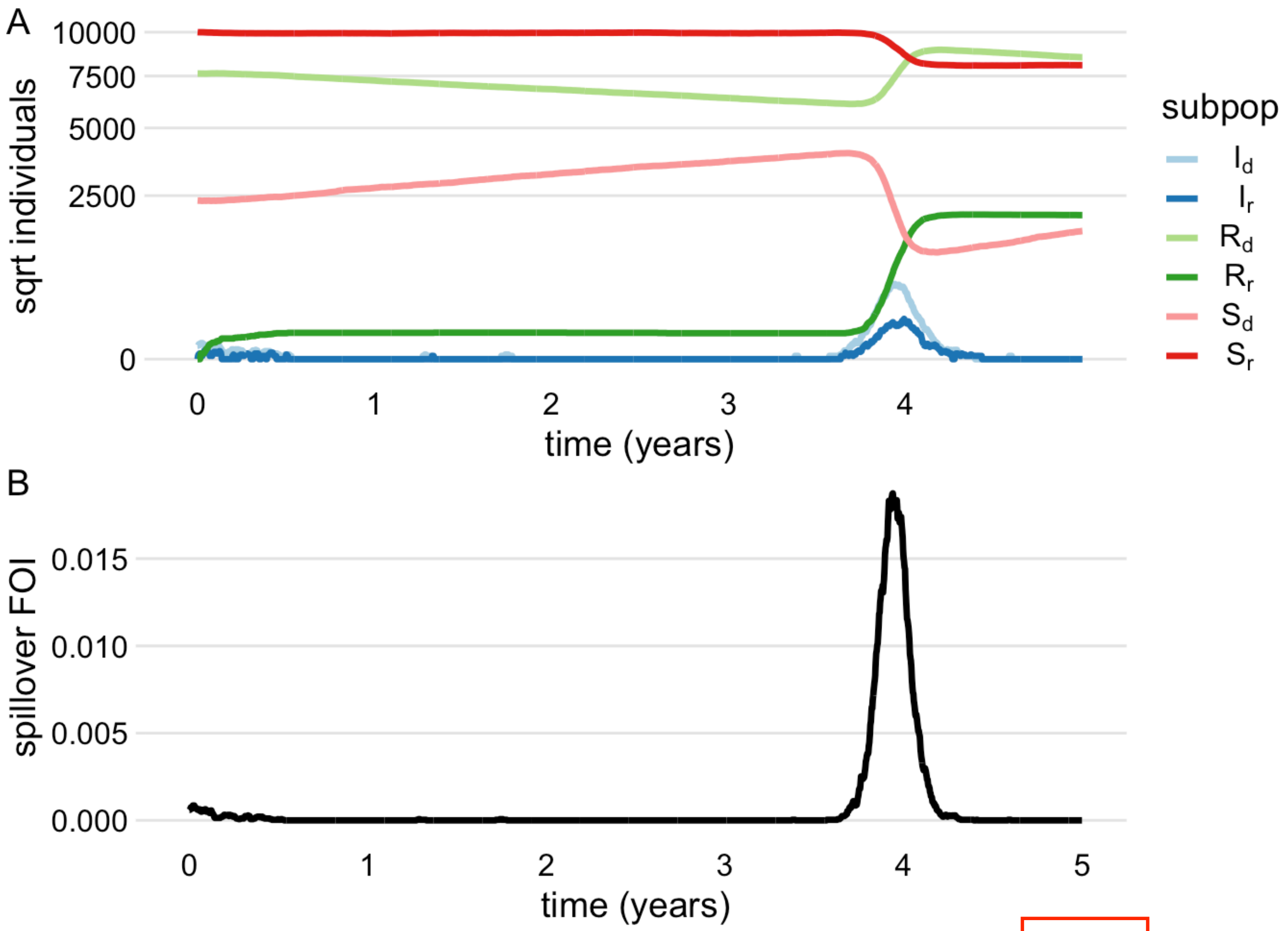

Figure S3: One random realization of dynamics for example system 2 ($R_{0_{r-r}} < 1$$R_{0_{r-r}} < 1$), with no interventions.

Impact of interventions

Example 1:

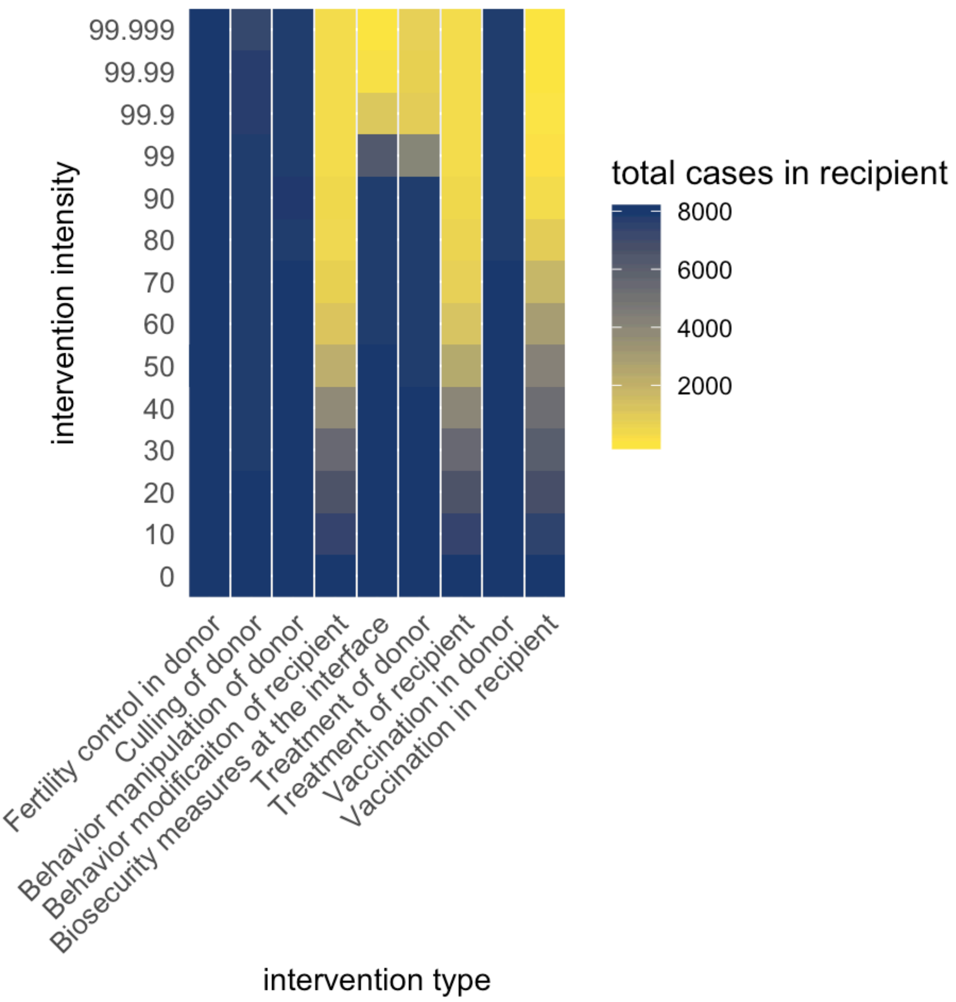

Figure S4: Total cases in the recipient population, as a function of the intensity (y-axis) of different ecological interventions (x-axis), for example system 1 ($R_{0_{r-r}} > 1 R_{0_{r-r}} > 1$). See Table S1 for parameter values used in this example.

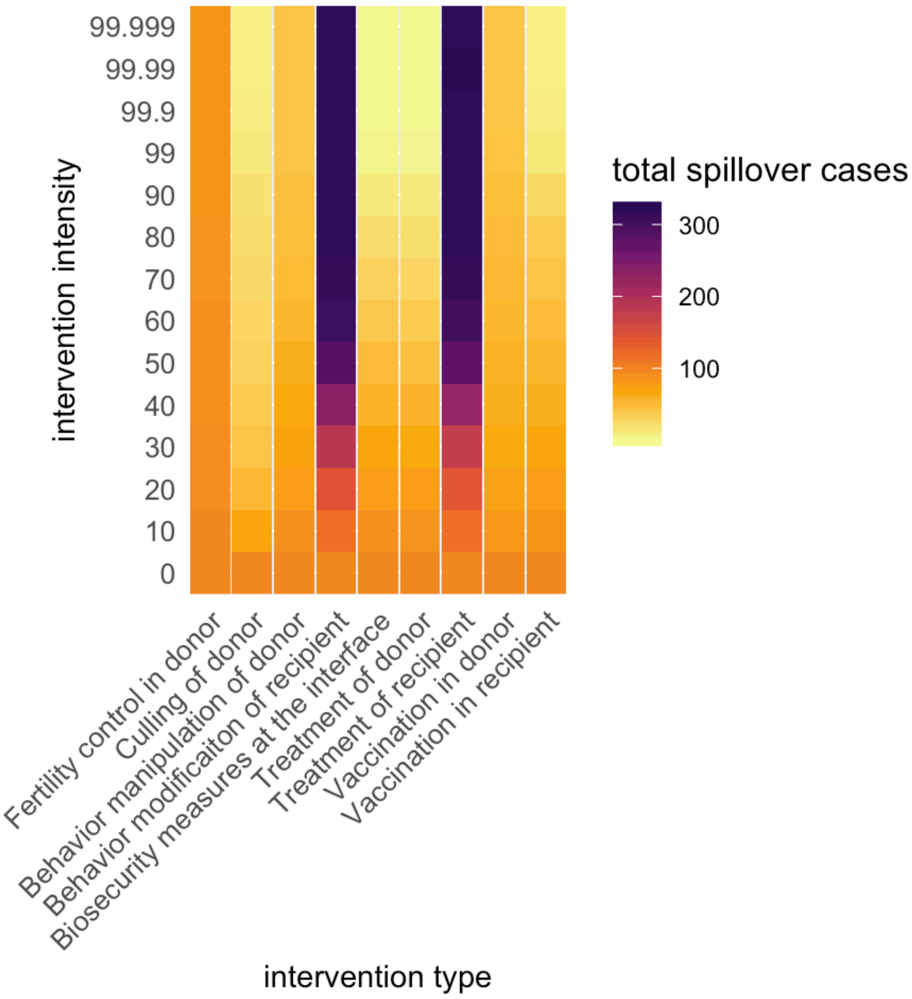

Figure S5: Number of spillover events (cases in the recipient population caused by donor-to-recipient transmission), as a function of the intensity (y-axis) of different ecological interventions (x-axis), for example system 1 $(R_{0_{r-r}} > 1 R_{0_{r-r}} > 1)$. Note that interventions that decrease recipient-to-recipient transmission without decreasing recipient susceptibility (i.e., behavior modification of the recipient, treatment of the recipient) can actually increase spillover relative to no management. This counterintuitive outcome occurs because these interventions reduce transmission within the recipient host, leaving more individuals susceptible to spillover. Thus, although there are more spillovers, there are fewer total cases in the recipient (Figure S4).

Example 2:

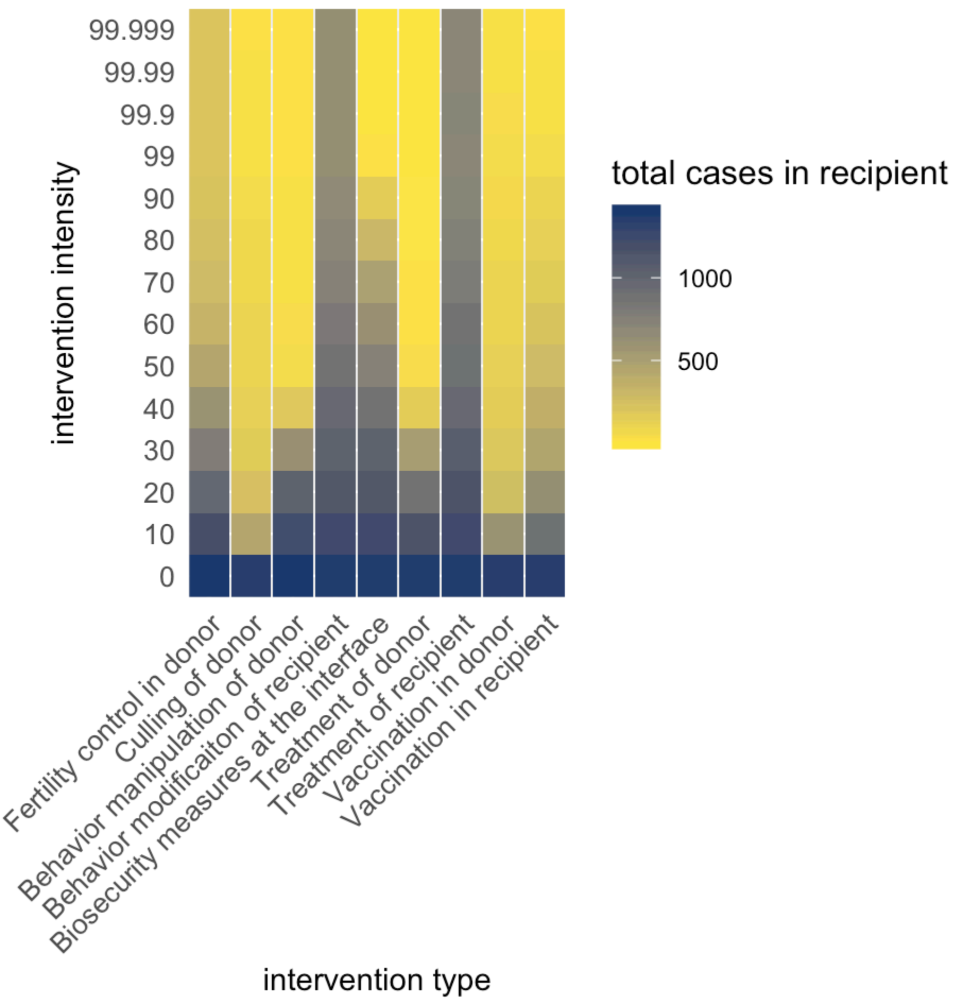

Figure S6: Total cases in the recipient population, as a function of the intensity (y-axis) of different ecological interventions (x-axis), for example system 2 ($R_{0_{r-r}} < 1 R_{0_{r-r}} < 1$). See Table S1 for parameter values used in this example.

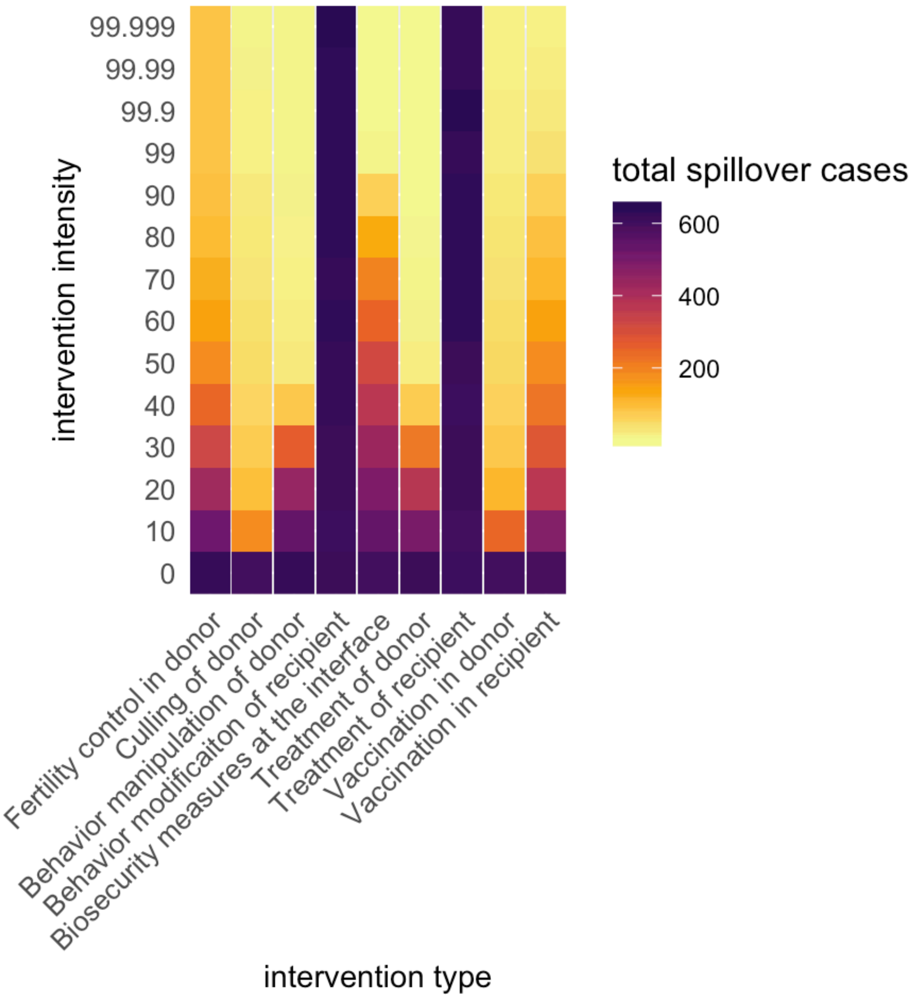

Figure S7: Number of spillover events (cases in the recipient population caused by donor-to-recipient transmission), as a function of the intensity (y-axis) of different ecological interventions (x-axis), for example system 2 ($R_{0_{r-r}} < 1 R_{0_{r-r}} < 1$).

References

[1] Abbey, H. (1952) An examination of the Reed-Frost theory of epidemics. Hum Biol 24, 201-233
[2] Fenton, A. and Pedersen, A.B. (2005) Community epidemiology framework for classifying disease threats. Emerg Infect Dis 11, 1815-1821
[3] Viana, M., et al. (2014) Assembling evidence for identifying reservoirs of infection. Trends Ecol Evol 29, 270-279
[4] R Core Team (2018). R: A language and environment for statistical computing. R Foundation for Statistical Computing, Vienna, Austria. URL https://www.R-project.org/ (https://www.R-project.org/).
[5] King, A.A., et al. (2018) pomp: Statistical Inference for Partially Observed Markov Processes (R package, version 1.19). https://CRAN.R-project.org/package=pomp (https://CRAN.R-project.org/package=pomp)
